# Inappropriate manipulation and drainage exacerbate post-operative pain and prolong the hospital stay after laparoscopic appendectomy for pediatric complicated appendicitis

**DOI:** 10.1186/s12893-021-01413-x

**Published:** 2021-12-25

**Authors:** Yi-Wen Tsai, Shin-Yi Lee, Jyun-Hong Jiang, Jiin-Haur Chuang

**Affiliations:** Department of Pediatric Surgery, Kaohsiung Chang Gung Memorial hospital, No. 123, Dapi Road, Niaosong District, Kaohsiung, 83301 Taiwan, R.O.C.

**Keywords:** Complicated appendicitis, Single-port laparoscopic appendectomy, Drainage, Visual analog scale, Length of stay

## Abstract

**Background:**

This study examined whether drain placement or not is associated with the postoperative outcomes of pediatric patients following trans-umbilical single-port laparoscopic appendectomy (TUSPLA) for complicated appendicitis.

**Methods:**

The medical records of pediatric patients undergoing TUSPLA for acute complicated appendicitis from January 2012 to September 2018 in Kaohsiung Chang Gung Memorial Hospital were reviewed retrospectively. They were classified according to whether they received passive drainage with a Penrose drain (Penrose group) (19), active drainage with a Jackson-Pratt drain with a vacuum bulb (JP group) (16), or no drain (non-drain group) (86). The postoperative outcomes of the three groups were compared.

**Results:**

Postoperative visual analog scale pain score was significantly higher in the non-drain group than in either the JP group or Penrose group. Patients in the Penrose group had a significantly longer postoperative hospital stay than those in the non-drain group and a higher rate of intra-abdominal abscess, while patients in the JP group had a significantly shorter postoperative hospital stay; moreover, no patient in JP group developed a postoperative intra-abdominal abscess.

**Conclusions:**

Compared to passive drainage with a Penrose drain or no drain, active drainage with a JP drain shorter the postoperative hospital stay and decreased the risk of postoperative intra-abdominal abscess.

## Background

Trans-umbilical single-port laparoscopic appendectomy (TUSPLA) was adopted by out hospital in July 2006, and had been performed in 827 of our pediatric patients by September 2018. TUSPLA provides satisfactory postoperative and cosmetic outcomes in the treatment of simple appendicitis. However, differences in postoperative outcomes according to intra-operative management, including the choice of drainage, in patients with complicated appendicitis have not been thoroughly assessed.

Complicated appendicitis is defined as the presence of a visible hole in the appendix, a fecalith in the abdomen detected intraoperatively, or an appendiceal perforation [1]. The development of a postoperative intra-abdominal abscess has been reported in 1.7–30% of patients with complicated appendicitis [2–6], but the advantages of drain tube placement after appendectomy remain unclear. After appendicitis surgery, the abdominal cavity must be cleaned by extensive irrigation and suction to remove intraabdominal pus or dirty ascites, and thus prevent intra-abdominal abscess formation. This is especially important when a drain tube will not be placed. Schlottmann et al. [3] reported no significant difference in the rate of intra-abdominal abscess formation between drain (passive drainage with a Blake silicone tube) and non-drain groups (14.2% and 8.9%, respectively). A Japanese study [7] showed that routine drainage did not confer advantages in terms of postoperative outcome or length of hospital stay in pediatric patients with complicated appendicitis. By contrast, both Pakula et al. [8] and Beek et al. [9] concluded that the use of a drain decreased the risk of intra-abdominal abscess formation.

Conflicting results have also been reported after laparoscopic appendectomy for complicated appendicitis [10, 11]. In these patients, the type of drain tube and distance from the abdominal wound to the most distal end of the tube play a role in the outcomes. The aim of this study was to assess whether the postoperative outcomes of patients undergoing TUSPLA for complicated appendicitis varied depending on whether extensive irrigation-suction without drainage or a drain tube allowing active or passive drainage was used.

## Method

### Patients

**Fig. 1:**
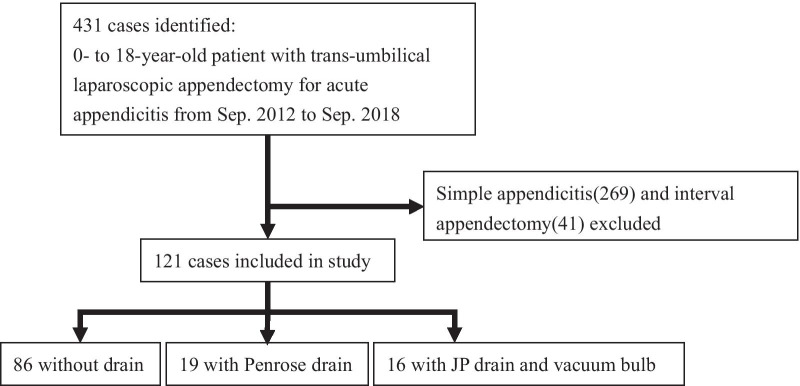
Study flow diagram

Since September 2012, all children aged 5–18 years diagnosed with acute appendicitis at our hospital (Kaohsiung Chang Gung Memorial Hospital) are treated by TUSPLA, without conversion to open appendectomy. For this study, the medical records of 431 TUSPLA patients treated at our hospital between September 2012 and September 2018 were retrospectively reviewed. The study included only those children with complicated appendicitis, defined as a visible hole in the appendix, a fecalith in the abdomen seen intraoperatively, or appendiceal perforation confirmed in the pathology report [1]. Patients with uncomplicated appendicitis (n = 269), and those treated by interval appendectomy (n = 41), were excluded. Thus, the study population consisted of 121 patients (Fig. 1) who were subsequently classified into one of three groups depending on whether they had received a drain, and on the type of drain tube used (no drain, Jackson-Pratt drain with a vacuum bulb [JP drain], or Penrose drain). Patient demographics, intraoperative details, postoperative visual analog scale (VAS) pain score, length of hospital stay, postoperative abscess formation, wound infection, and ileus were recorded and analyzed.

### Surgical technique

All patients received a single dose of cefazolin, gentamycin, and metronidazole preoperatively. TUSPLA was performed as described in our previous publication [12]. After appendectomy, blood, pus, abscess, and fecal material were suctioned out; the suction irrigator was drawn out from the port to prevent the accumulation of residual fluid in the peritoneal cavity.

Most of the patients in the early phase of the study period did not receive a drain after appendectomy. The responsible surgeon believed that extensive irrigation of the abdomen with 2500–3000 mL of lactate-Ringer solution, followed by repeated suctioning until the peritoneal fluid was clear, was sufficient to prevent postoperative abscess formation. However, this was not always the case, so the surgeon later chose to place either a Penrose drain (silastic Penrose drain, 6 mm in wide) or JP drain (Jackson-Pratt® flat perforated drain, 7 mm in tube diameter) after irrigation of the abdominal cavity with 500–1000 mL of lactate-Ringer solution followed by suctioning. The drain was placed in the pelvic cavity and exited the abdomen through the umbilical wound; one stich was sutured on the facia to approximate the umbilical exit site so that the drain was not prone to slip out. The umbilical fascia and subcuticular layer of the skin were closed with 5-0 polydioxanone sutures.

### Postoperative management

After surgery, early feeding and ambulation were encouraged in all patients. Intravenous fluids were discontinued once the patient’s appetite had recovered. A standardized analgesic regimen was administered for pain relief, including oral acetaminophen and ibuprofen every 6 h and 0.1 mg morphine/kg, administered intramuscularly, if the VAS pain score was above 4.

In the Penrose drain group, the drain was removed after the dressing had been infiltrated with a minimal amount of clear fluid. In the JP drain group, the drain was removed when the amount of clear fluid drained was < 50 mL/day.

All patients received intravenous antibiotics (cefazolin, gentamycin, and metronidazole) until discharge from the hospital. Patients were discharged when normal diet and daily activities resumed in the absence of fever and abdominal pain, and after removal of the tube. Discharged patients were prescribed oral Augmentin (amoxicillin and clavulanate potassium) for 7 days. During the out-patient follow-up, if the patient continued to experience right lower abdominal pain without fever, oral antibiotics were continued for another week. Ultrasonography was performed by a pediatric radiologist in patients suspected of having an intra-abdominal abscess. Patients with an intra-abdominal abscess were admitted for 5–7 days of intravenous antibiotics therapy. Image-guided drainage evaluation, using either sonography or computed tomography, was done when antibiotic treatment was ineffective, or when the abscess was > 4 cm in diameter. Wound infections were clinically evaluated by the surgeon in terms of symptoms including hyperemia, purulent discharge, swelling, and induration of the wound. Ileus was defined according to the clinical history, and in the presence of abnormal physical and radiological findings, including a distended abdomen or prolonged absence of flatus.

### Statistical analysis

The data were analyzed using SPSS software (ver. 24.0; SPSS, Inc., Chicago, IL, USA). Continuous variables were compared using the Kruskal–Wallis test, and categorical variables using the chi-squared test. A *p* value ≤ 0.05 was defined as statistically significant. Multivariate analysis was performed using a logistic regression model, with the results expressed as odds ratios (ORs) and 95% confidence intervals (CIs). To identify risks factors, pre- and intra-operative variables identified as significant in the univariate analysis were entered into the multivariate logistic regression model with likelihood ratio forward selection.

## Results

The preoperative characteristics and operative findings of the 121 patients are shown in Table 1. The number of the patients in the non-drain, Penrose drain, and JP drain group was 86, 19, and 16, respectively. There was no significant difference among groups in age, sex, mean white blood cell count, percentage of segment and lymphocytes, level of C-reactive protein, ascites, or operative time.

**Table 1:** Pre-operative characteristics and operative findings, according to univariate analysis

Group	Non-drain (N = 86)	Penrose drain (N = 19)	JP drain (N = 16)	*p* value^†^
Age (yrs.)	11.5 ± 3.59	11.0 ± 3.92	9.3 ± 3.64	0.077
Male: female	56:30	13:6	9:7	0.735
Body weight	43.27 ± 17.08	43.39 ± 21.16	34.49 ± 15.53	0.181
WBC (1000 μ/dL)	17.62 ± 5.77	17.28 ± 5.67	13.55 ± 6.44	0.091
Band (%)	0.63 ± 1.41	0.9 ± 1.39	2.53 ± 9.47	0.739
Seg. (%)	83.75 ± 6.02	84.56 ± 4.77	79.89 ± 14.52	0.872
CRP	102.51 ± 88.19	140.67 ± 127.61	128.45 ± 108.08	0.707
Operation finding				
Diffuse purulent ascites	48 (55.81)	14 (73.68)	13 (81.25)	0.082
Fecalith	54 (62.79)	10 (53.63)	9(56.25)	0.671
Abscess formation	45 (52.33)	8 (42.11)	6 (37.50)	0.452
Operation time (min)	107.0 ± 45.1	130.2 ± 50.0	91.9 ± 29.4	0.060

*WBC *white blood cell count, *Seg. *segment percent of WBC, *Band *band form percent of WBC, *CRP* serum C-reactive protein, *JP drain* Jackson-Pratt drain with vacuum bulb

*p value < 0.05 indicate statistical significance among three group

^†^Comparison among JP drain group, Penrose drain group, and non-drain group

The postoperative outcomes after univariate analysis are summarized in Table 2. The VAS pain score on postoperative day 1 (POD1) was significantly higher in the non-drain group (4.1 ± 1.25) than in either the JP drain group (2.3 ± 0.6, *p *< 0.001) or Penrose drain group (3.1 ± 1.5, *p *= 0.013) (Table 3). There was no difference among the three groups on POD3 (2.1 ± 0.76, 1.9 ± 0.50, and 2.1 ±  0.54, respectively, *p *= 0.243; Table 2).

**Table 2:** Post-operative results and outcome, according to univariate analysis

Group	Non-drain (N = 86)	Penrose drain (N = 19)	JP drain (N = 16)	*p* value^†^
Post-operative LOS (days)	5.22 ± 1.25	6.26 ± 1.67	4.5 ± 1.70	< 0.001*
VAS on POD1	4.1 ± 1.25	3.1 ± 1.50	2.3 ± 0.60	< 0.001*
VAS on POD3	2.1 ± 0.76	2.1 ± 0.54	1.9 ± 0.50	0.243
Days until full diet achieved (days)	3.6 ± 1.44	4.1 ± 1.56	2.6 ± 0.89	0.007*
Prolonged oral antibiotics	11 (12.79)	2 (10.53)	3 (18.75)	0.700
Post-operative abscess, n (%)	5 (5.81)	4 (21.05)	0 (0)	0.056
IV antibiotics	3 (3.49)	3 (15.79)	0 (0)	0.098
Drainage	2 (2.33)	1 (5.26)	0 (0)	0.645
Ileus, n (%)	5 (5.81)	1 (5.26)	1 (6.25)	0.598
Wound infection, n (%)	1 (1.16)	1 (5.26)	0 (0)	0.497

Days until full diet achieved = post-operative days until full diet achieved

Prolong oral antibiotics = oral antibiotics for 1 week after first visit of out-patient department

IV antibiotics = readmission for intravenous form antibiotics for about 5 to 7 days

Drainage = post-operative abscess that need drainage and re-admission for intravenous form antibiotics

*LOS *length of hospital stay, *VAS* visual analog scale, *POD* post-operative day

*p value < 0.05 indicate statistical significance among three group

^†^Comparison among JP drain group, Penrose drain group, and non-drain group

**Table 3:** Multivariate logistic regression of outcome: VAS POD1 (VAS > 4)

Variables	*p* value	Odd ratio	95% CI	
			Lower	Upper
Retrocecal appendicitis	0.027*	0.249	0.073	0.853
JP drain	0.000*	0.013	0.002	0.104
Penrose drain	0.002*	0.175	0.058	0.533

*VAS POD1 *visual analog score on post-operative day 1

*p value < 0.05 indicate statistical significance

The time to resumption of a full diet after surgery was shorter in the JP drain group than in the non-drain group (2.6 ± 0.89 vs. 3.6 ± 1.44, *p *= 0.021). The postoperative length of hospital stay was shorter in the JP drain group, but longer in the Penrose drain group, than in the non-drain group (4.50 ± 1.70 vs. 5.22 ± 1.25 days, *p *= 0.027; 6.26 ± 1.52 vs. 5.22 ± 1.25 days, *p *= 0.029, respectively). According to the results of the multivariate analysis, the Penrose drain, but not the JP drain, prolonged the postoperative length of hospital stay (adjusted OR 2.998, 95% CI 1.015–8.857, *p *= 0.047; Table 4).

**Table 4:** Multivariate logistic regression of outcome: length of hospital stay (> 5 days)

Variables	*p* value	Odd ratio	95% CI	
			Lower	Upper
Abscess	0.015*	2.773	1.220	6.303
JP drain	0.145	0.311	0.064	1.497
Penrose drain	0.047*	2.998	1.015	8.857

*Abscess* intra-operative finding of abscess formation

*p value < 0.05 indicate statistical significance

A postoperative intra-abdominal abscess developed in 5 of 86 (5.8%) patients in the non-drain group, 4 of 19 (21.1%) patients in the Penrose drain group, and no (0%) patients in the JP drain group, although the difference between the groups not significant (*p *= 0.056). While no patients in the JP drain group had an intra-abdominal abscess, the small size of this group at least in part accounted for the lack of a significant difference compared to the non-drain group. A Penrose drain increased the risk of postoperative abscess (adjusted OR 4.629, 95% CI 1.1065–19.379, *p *= 0.036). In the non-drain group, two patients (2.33%) had image-guided trans-abdominal drainage; in the Penrose drainage group, one patient (5.26%) had CT-guided trans-rectal drainage.

Wound infection occurred in one patient in the non-drain group (1.16%) and one in the Penrose drain group (5.26%); the rate was not significantly different. Both patients were managed in the outpatient department with oral antibiotics and regular dressing changes. Ileus occurred in five patients in the non-drain group (5.81%), one patient in the Penrose drain group (5.26%), and one patient in the JP drain group (6.25%). The difference among groups was not significant. All seven patients were treated conservatively with intravenous fluid hydration and recovered uneventfully.

The duration of follow-up in this study is 2 years. There is no long term complication such as incisional herniation or sinus formation, both in JP drain group and Penrose drain group.

## Discussion

Postoperative pain is both somatic, originating from the surgical incision, and visceral, due to injury to the peritoneum and intra-abdominal structures [13]. Visceral postoperative pain arises from the peritoneal incision [13–15], peritoneal closure [16], traction of the peritoneum, pressure from extensive irrigation with isotonic saline [17], and intraperitoneal inflammation, all of which cause injury to the peritoneum [18] and thus postoperative pain [19, 20]. Extensive irrigation and manipulation of the peritoneum to avoid placement of a drain was associated with a significantly higher pain score on POD1 in our study. Intraperitoneal inflammation and postoperative pain may increase surgical stress [21, 22] and thus influence the surgical outcome [23, 24]. To reduce both surgical stress and postoperative pain [25], and to minimize inflammation, extensive irrigation should be avoided. This will also diminish the duration of postoperative ileus and decrease patient morbidity [21, 22, 26].

Previous studies demonstrated that extensive irrigation to avoid placement of a drain is ineffective in reducing the risk of postoperative abdominal abscess [27–29]. In our study, the use of an appropriate drain reduced not only the length of the hospital stay, but also the risk of postoperative abscess. Complicated appendicitis requires active drainage, as passive drainage is insufficient to remove turbid fluid in the peritoneal cavity. Two studies [8, 9] reported positive effects with the use of a closed-system peritoneal drain, such as the JP drain, including a lower risk of postoperative intra-abdominal abscess, as well as reduced rates of re-intervention and readmission, in patients with complicated appendicitis treated by laparoscopic appendectomy. However, those findings were not supported by another study [30]. Castro et al. assessed the outcomes of pediatric patients who had undergone laparoscopic appendectomy for perforated appendicitis [6]. The results showed that, compared to the non-drain group, patients with a passive drain had longer-duration antibiotics therapy (6.61 vs. 7.51 days) and a longer postoperative hospital stay (9.2 vs. 11.55 days), whereas there was no difference in the rate of intra-abdominal abscess or wound infection. Similar results were obtained by Schlottmann et al. [3]. In the study by Allemman et al. [31], routine drainage of the abdominal cavity for complicated appendicitis was associated with a higher rate of wound infection, but the type of drain used in that study was not reported. Whether the drain type affects the outcome of patient with complicated appendicitis treated by appendectomy is unknown.

The closed-suction system in the JP drain provides active drainage, as the negative pressure allows evacuation of pus or purulent ascites [32]. The Penrose drain makes use of passive drainage through an open-system that relies on natural pressure gradients or capillary action, as well as gravity flow, muscle contraction, and overflow [33, 34]. The exit site of the drain in our TUSPLA patients was the umbilical incision wound, in contrast to right lower quadrant port incision used in conventional laparoscopic appendectomy. An exit site in the umbilicus provides better cosmetic outcomes, but the distance to the pelvic cavity is longer such that passive drainage is inefficient. In addition, retrograde migration of bacteria along the tract of the Penrose drain may increase the risk of descending infection [33, 35, 36], and thus abscess formation [33]. Early removal of an abdominal drain was shown to increase the risk of intra-abdominal abscess [36]. In our practice, the drain is removed once the criteria described for the JP drain group have been met, as our experience has shown that this not only lowers the risk of intra-abdominal abscess formation but also shortens the hospital stay.

Incisional hernia is a concern for placing drain from umbilicus. Our short term placing a tube in the umbilical exit site is similar to placing a trocar through the umbilicus for laparoscopic procedures. According to the literature, the trocar site hernia occurs predominantly at the umbilical site and especially when greater than 10mm in facia defect [37]. In our practice, we used Jackson-Pratt® flat perforated drain and the tube size was 7 mm in diameter. After placing the drain, we usually sutured one stich to approximate the umbilical exit site so that the drain was not prone to slip out. After removing the tube, the facia defect was small and the risk of incisional herniation was thus low.

Among the limitations of our study were the small number of patients in two of the three groups and the retrospective design, such that selection bias cannot be ruled out. However, our findings could still guide pediatric surgeons in the selection of an appropriate drain tube in pediatric patients after appendectomy for complicated appendicitis.

## Conclusions

Extensive peritoneal lavage without a drain may exacerbate postoperative pain after laparoscopic appendectomy for complicated appendicitis. Active drainage with a JP drain shortens the hospital stay, decreases the risk of post-operative intra-abdominal abscess, and is associated with significantly less pain immediately after surgery. Passive drainage is not advised in TUSPLA in patients with complicated appendicitis.

## Data Availability

The datasets generated and analysed during the current study are not publicly available because the data was extracted from electronic medical chart, retrospectively but are available from the corresponding author on reasonable request.
